# Current Advances in Molecular Mechanisms and Physiological Basis of Panicle Degeneration in Rice

**DOI:** 10.3390/ijms20071613

**Published:** 2019-04-01

**Authors:** Asif Ali, Peizhou Xu, Asad Riaz, Xianjun Wu

**Affiliations:** Key Laboratory of Crop Genetic Resources and Genetic Improvement, Ministry of Education, Institute of Rice Research, Sichuan Agricultural University, Chengdu 611130, China; asifalikalas@foxmail.com (A.A.); xpzhxj@163.com (P.X.); asad.riaz76@gmail.com (A.R.)

**Keywords:** panicle degeneration, source limitation, programmed cell death, phytohormones

## Abstract

Panicle degeneration, also known as panicle abortion, is a serious defect and causes heavy losses to reproductive yield in cereals. Several mutants have been reported to display the phenotype of spikelet abortion in rice. Recent findings have resulted in significant breakthroughs, but comprehensive understanding about the molecular pathways and physiological basis of panicle degeneration still remain a dilemma. In this review, we have summarized all the responsible genes and mechanisms underlying the panicle development with a special focus on degeneration. Here, we hypothesized a model by using knowledge and coherent logic in order to understand the molecular regulation of panicle degeneration. In addition to this, we included all the previous discoveries, schools of thoughts, ancient working theories, and crosstalk of phytohormones and provided new insights for future studies.

## 1. Introduction

Rice is an important food crop that supplies rations for over one-half of the world’s population [1]. The cognitive traits of yield are largely affected by genetic, biotic, and abiotic factors [2]. The occurrence of degenerated spikelets is a common phenomenon that causes a significant reduction in yield. The genetic factors and the molecular mechanisms of panicle degeneration are current safeties of several molecular biologists to sustain rice productivity. Even though the recent findings of panicle degeneration have provided new clues about its regulation, there is a dire need to build up a comprehensive understanding. A transcriptome study revealed that 357 of 22,000 genes played a differential role in panicle development, and it suggested that many genes are not yet identified. [3]. In this regard, the aberrant or deficient panicle mutants are key concepts for the understanding of the regulation of panicle development. 

The number of differentiated spikelets is a major contributor to grain yield, and degenerated spikelets account for up to 20% of the differentiated spikelets [4]. The spikelet degeneration rate depends mostly upon the type of cultivar and it ranges from 50–60% under extreme weather conditions [4,5]. The panicle development is mainly categorized into two stages, i.e., panicle initiation and panicle elongation. In most of the studies, spikelet degeneration has been reported during the panicle elongation stage. For example, an invasion of any stress during meiosis, could be incurable to pursue the development of floral organs that results in degeneration [6]. Studies have also revealed numerous genetic bases of degeneration in wheat, maize, and rice [7,8,9]. Despite the discovery of several mutants, for example, *psd* (*panicle and spikelet degeneration)* and *paa-hwa* (*panicle apical abortion*), the physiological clarifications of the causal mechanism of degeneration could not be explored [10,11]. Most of these mutants were reported to bear an SNP (single nucleotide polymorphism) in their respective candidate gene. Similarly, a series of QTLs (quantitative trait loci), for example, *qPAA3* (*panicle apical abortion*), *qPAA4*, *qPAA5*, and *qPAA8* have also been reported in rice, which display the panicle abortion due to excessive accumulation of (H_2_O_2_) hydrogen peroxide [12]. 

In order to explain the basis of panicle degeneration, there are two classical theories at work [13]. According to the first theory, “resource limitation” is a typical reason for differentiated spikelets to degenerate, as the nutrient supply to inferior spikelets becomes insufficient under the stress condition [14,15]. Low occurrence of non-structural carbohydrates causes spikelets to starve under the strong competition of food, and as a result inferior spikelets are forced to degenerate [16]. Unfortunately, the resource limitation theory only addresses some of the cases that have been elucidated in recent studies as degeneration has also been reported under the favorable conditions [17,18]. According to the second theory, panicle degeneration occurs due to a “self-organization” where plants render the spikelet to degenerate through an endogenous catalytic process. Random migration of food to superior sinks leads to the degeneration of inferior sinks [19]. The self-organization theory does not explain the mechanism of apical dominance, that is, wheat degeneration appears at both distal ends, whereas in maize and rice degeneration it mostly borders to the apical position. The evidence is not convincing enough to support the assumptions that were proposed in both theories [7,9,12]. It is worthwhile to note that the structure of rice inflorescence is significantly different from that of maize, wheat, and *Arabidopsis*. One spikelet of inflorescence is equal to 7–10 florets in wheat, two florets in maize, and only one floret in rice [20]. On the basis of morphology and structure of inflorescence, the mechanism and extent of degeneration may vary. Although, some basic physiological mechanisms that regulate the panicle architecture are similar in monocots and dicots, for example, the function of the AP2 gene family in rice has been reported to play a similar role in maize and *Arabidopsis* [21]. In addition, a comparative analysis of rice panicle morphology with maize, *Arabidopsis*, and other model plants will uncover the conserved and divergent regulatory pathways controlling plant reproduction. 

## 2. Control of Panicle Development by Meristems Organization

The panicle developmental process is accomplished when vegetative meristems are changed to the reproductive meristems [22]. A molecular circuit of panicle development in rice is regulated through a complex network of genes that initiates with the change of shoot meristems to the progression of axillary meristems (AMs). Afterwards, AMs are derived by the genes that control meristem transition to (FMs) floral meristems [22]. The network of meristem controlling genes is largely affected by abiotic stresses and several endogenous cues [23]. Phytohormones, especially auxin and cytokinin, regulate the meristems initiation [24]. The dysregulation of meristem controlling genes mainly lead to production of abnormal development of anthers due to obstructions in the meiosis and cell cycle related processes [25]. We categorized the meristem organization into the following phases in order to make it easier to understand.

### 2.1. Axillary Meristem (Number/Size)

Development of panicle begins with the formation of primordia [26]. The developmental transition of shoot apical meristem (SAM) to reproductive meristem and subsequently inflorescence development is largely regulated by several endogenous and environmental signals. CLAVATA pathway genes functions have been conserved in monocots and dicots, which play their role in signaling for the development of SAMs [27]. *LAX1 (LAX PANICLE 1)* is the homolog of *BA1 (BARREN STALK 1)* in maize [9] and encoded a transcription factor containing bHLH (BASIC HELIX LOOP HELIX) domain that is required for formation of AMs in rice [28]. *LAX1* controlled the inflorescence identity of terminal meristems and interacted with *LAX2* [29]. *LAX2* regulated the maintenance of AM for both vegetative and reproductive branching. The double mutant of *lax1lax2* displayed the phenotype of reduced branching. Similarly, *SPA (SMALL PANICLE)* also played an overlapping role with that of *LAX1* and controlled the branching of shoots by AMs [30]. *MOC1 (MONOCULM 1)* promoted the tillering of buds by initiating axillary buds. The *moc* mutant could not be developed with all lateral tillers but could only be developed with one, due to defects in the initiation of inflorescence meristems (IMs) differentiation [31]. *OsH1 (ORYZA STAIVA HOMEOBOX 1)* is the homolog of *KN1 (KOTTED 1)* in maize, [32] and highly expressed in the cotyledon and IMs. The *osh1* mutant produced fewer spikelets that resulted into smaller inflorescence [33].

### 2.2. Meristem Transition Controlling Genes

A change of indeterminate meristem fate to determinate meristem is essential for the reproductive architecture of the plant. *ASP1 (ABERRANT SPIKELET AND PANICLE 1)* is the homolog of the *TOPLESS (TPL)* gene in *Arabidopsis* [34] that encoded a co-repressor involved in auxin signaling pathway [35]. The *asp1* mutant showed pleiotropic defects in branching, spikelet, and phyllotaxy associated with the auxin signaling. Furthermore, its role in the control of meristem transition still needs more clarification. *FZP (FRIZZY PANICLE)* is a homolog of *BD1 (BRANCHED SILKLESS 1)* in maize [36], and it controlled the transition of meristem identity by averting the AMs formation through an ERF (ETHYLENE RESPONSE FACTOR) in rice [37]. Strong alleles in the *fzp* mutant revealed the presence of branches instead of spikelets, whereas weak alleles displayed the spikelets at the apices of branches. During the ear development of maize, *BD1* regulated the transformation of AMs to FMs [38]. Overexpression of miR172b delayed the transition of AMs to FMs and caused the spikelet indeterminacy [21]. *TAW1 (TAWAWA1)* encoded a nuclear protein and worked in the upstream of MADS-box genes to regulate the phase transition by promoting the activity of IMs [39]. The *tawawa-1* is a gain-of-function mutant that exhibited the higher activity of IMs and displayed an increased number of spikelets. *RCN1 (REDUCED CULM NUMBER 1)* and *RCN2* are homologs of *TF1 (TERMINAL FLOWER 1)* in *Arabidopsis*, [40] and adjusted the panicle morphology by governing the period of phase transition [41]. *APO1 (ABERRANT PANICLE ORGANIZATION 1)* regulated the identity of IMs and played a temporal role in the development of vegetative and reproductive phenotypes [42]. 

### 2.3. Floral Meristems Controlling Genes

FMs are required to initiate the growth and development of male/female reproductive parts. *APO1* is the homolog of *UFO (UNUSUAL FLORAL ORGAN)* in *Arabidopsis* that encodes an F-box protein and regulates the FMs [43]. *APO1* positively regulated the expression of C-class homeotic genes, and their overexpression displayed an increased panicle size and leaf number [44]. *APO2* displayed small panicles by interacting with *APO1* and prevented the transition of IMs to FMs [45].

## 3. Regulation of Inflorescence Development by Phytohormones

Phytohormones are produced within the body of plants and transported to the sites, where they are required to regulate the variety of physiological functions including panicle development. At present cytokinin (CTK), gibberellins (GA), brassinosteroid (BR), auxin, and abscisic acid (ABA) are reported to play an essential role in the panicle development [46]. Some other phytohormones, such as the higher rate of ethylene, have also been correlated with the degree of abortion in maize [47]. In addition to phytohormones, some secondary metabolites, such as peptides and strigolactones, also regulated the developmental events in panicle [46]. 

### 3.1. Role of CTK in Panicle

CTK promotes cell division, and it regulated the activity and the size of meristem that can affect the panicle morphology, directly or indirectly [48]. It is evident that changes in the metabolism and signaling pathway of CTK had a prodigious impact on the development of an inflorescence [49]. *GN1 (GRAIN NUMBR 1)* is a gene for CKX2 (CYTOKININ DEHYDROGENASE/OXIDASE) and its reduced expression caused the accumulation of CTK in the panicle that resulted in higher grain yield. *LP (LARGER PANICLE)* encodes an F-box protein and induced the expression of *OsCKX2*, which can modulate the level of CTK [50]. *LOG (LONELY GUY)* encodes a cytokinin-activating enzyme, and its loss of function led to pre-mature termination of meristem development [24]. *DEP1 (DENSE AND ERECT PANICLE 1*, also called *qPE9-1*) regulated the expression of *OsCKX2* that is positively correlated with the meristematic and cell proliferation activity [51]. 

### 3.2. Role of GA in Panicle

GA has been reported to play an essential role in reproductive development. A GA-deficient mutant has revealed defects in anther morphology due to the poor development of pollen exine and ubisch bodies [52]. Application of GA_3_ alleviated the symptoms of degeneration under a saline stress condition [53]. *GNP1 (GRAIN NUMBER PER PANICLE 1)* increased the CTK action in meristem and promoted the yield by controlling the expression of GA20ox1 (GIBBERELLIN 20 OXIDASE 1) [54]. Furthermore, it is evident from the previous studies that CTK and GA played an antagonistic role during the reproductive meristem phase. Hereafter, we can deduct from the findings of previous studies that superior spikelets (strong sinks) possessed the higher contents of indole 3-acetic acid (IAA) and GA, which promoted the cell division and elongation of spikelets, while CTK and ethylene inhibited the development of superior spikelets and made the inferior spikelets more responsive.

### 3.3. Role of BR in Panicle

BRs are believed to play a variety of developmental roles, of which vascular differentiation, production of flowers, pollen tube, and protein biosynthesis are relevant to panicle morphology [55]. OsmiR397 has been found to increase the panicle branching by mediating the expression of BR [56]. The levels of homobrassinolide and epicastasterone have been found to negatively correlate with the abortion of spikelets [57]. BRs and IAA have been reported to induce the higher levels of salicylic acid during heat stress that mitigated the degeneration of spikelets in rice [58]. The direct effect of BRs on the panicle degeneration has not yet been reported.

### 3.4. Role of Auxin in Panicle

Auxin is an important growth regulator that controlled the panicle development by the initiation of AMs [59]. The maximum quantity of auxin that is transported to sink is used during the formation of primordium [60]. *BA1* is a homolog of *LAX1* and it has been reported to control the AMs formation by auxin transport [9,61]. The *ASP1* gene regulated the panicle morphology by auxin crosstalk [35]. A study has hypothesized that the low expression of IAA caused the malformations in panicle development that ultimately led to an abortion of spikelets [62]. The expression of *OsIAA20* was reported to play an important role in the formation of axillary buds in *asp1* mutant [35]. The IAA contents have been found to be varied in different positions of panicle and before and after the pollination [63]. Decreased transport of IAA to the inferior sink rather than that of the superior, act as a signal for the degeneration of sink [64]. It is commonly believed that spikelet degeneration occurs as a result of apical dominance. According to the resource limitation theory, export of IAA to latterly developed spikelets become insufficient than that of early developed spikelets. The depressed level of IAA to subordinate sinks caused the degeneration of fruit or spikelet [64]. Therefore, the spray of IAA to rice seedlings could not alleviate the panicle degeneration, and the higher concentrations of IAA also promoted the panicle degeneration [6,65]. This paradox behavior of IAA poses difficulties in terms of the apical dominance hypothesis, and as yet further evidence is needed to reach a final conclusion.

### 3.5. Role of ABA in Panicle

ABA is one of the important phytohormones that affects many aspects of plant biology. It has been reported to reduce the sterility of rice spikelets by the extraneous application of ABA with ethylene as the releasing agent [66]. A study has demonstrated the antagonistic effect of ABA and ethylene on grain filling and cell division [57]. Furthermore, upper position panicles contained a higher level of ABA, hence they are more prone to degeneration [7]. ABA and ethylene have been found to negatively correlate with the expression of the starch synthesis gene, thereby reducing the source to sink ratio [67]. ABA and ethylene were regarded as inhibitory growth regulators [68] because elevated levels of ABA and ethylene caused the abortion of inferior spikelets [67,68]. Although, the number of spikelets were increased under water stress due to higher level of ABA and ethylene [66]. The role of ABA in the panicle degeneration is not clear yet, it can be associated with the antagonistic effect of ABA and ethylene [66].

## 4. Inflorescence Degeneration Occurs Due to Limitation of Source Transportation

The carbohydrates, phospholipids, antioxidants are required for normal development of spikelets. During stress conditions, contents of antioxidants and non-structural carbohydrates have been reported to decrease and ultimately cause aberrations in panicle development [69]. *SP1 (SHORT PANICLE 1)* encoded a PTR (PEPTIDE TRANSPORTER) that maintained the panicle size by regulating the transport of an unknown substrate [65]. PTR family of proteins was involved in the transportation of different assimilates, including nitrate to the different parts of plants [70]. *OsALMT7 (ALUMINUM ACTIVATED MALATE TRANSPORTER 7)* transported the malate to panicle in order to maintain the panicle size in rice. A mutant deficient of *OsALMT7* revealed the apical spikelet degeneration that was accompanied due to cell death in the apical spikelets [71]. *OsC6* encoded an LTP (LIPID TRANSFER PROTEIN) that was essential for transport of lipids to the pollen exine. A mutant with the loss of function of *OsC6* displayed the abnormal post-meiotic development of anthers and orbicules [25]. *TUT1* (*TUTOU1)* encoded a SCAR (SUPPRESSOR OF CAMP RECEPTOR) protein that mediated the actin organization complex to maintain the panicle development in rice. *Tut-1* mutant revealed the degeneration of apical spikelets and poor development of pollen grains due to malformation of cytoskeleton and actin organization. The fatty acid profile of *tut1* mutant was deficient for eicosenoic acid (C20) and docosanoic acid (C22), revealing that the altered metabolism of long chain fatty acids was also responsible for *tut1* phenotype [8]. The characterization of *OsC6, OsCIPK31* and *SPL6* support the source limitation theory more than that of the self-organization theory. The prevalence of degeneration in lower spikelets further revealed that self-organization theory tends to falsify the concept of apical dominance. Panicle degeneration has also been reported under a good supply of water and nutrients that reveals that the source limitation theory accounts partly for panicle degeneration [4,72,73]. These results imply that both theories partly meet the basis of panicle degeneration and that further evidence is needed to reach a final conclusion.

## 5. Role of Transporting Tissues

Vascular bundles transport the food and water from the source to the sink, and their blockage due to any factor can cause the partial or complete spikelet degeneration. In addition to translocation of food, vascular bundles also provide mechanical support and their number is used to differentiate between japonica and indica cultivars [74]. A *paa1019* mutant paraffin sectioning revealed the blockage and destruction of vascular bundle cells [5]. A *paab1* mutant was also found defective in the translocation of malate into phloem parenchymatous cells of an apical spikelet and displayed the panicle degeneration [71]. *EP* (*ERECT PANICLE*) encoded a novel protein of endoplasmic reticulum and revealed erect panicle morphology due to an increased number of vascular bundles [75]. Similarly, *DEP1* and *DEP2* affected the cell proliferation rate and were considered ideal for rice breeding to increase the yield [76,77]. Similar to EP, *DEP2* affected the formation and number of vascular bundles, and therefore they have been widely researched to increase the yield of rice [78]. However, further specific molecular control of vascular bundles should be considered in future studies.

## 6. Role of Programmed Cell Death (PCD)

PCD is an integral response of plant against pathogens or abiotic stress. PCD was normally induced to any entity due to a stimulus and caused the destruction of cells [79]. The activation of the immune system to destroy the cells was stimulated by a variety of caspases and proteases [80]. Although the mechanism of PCD is not well understood, it can be broadly categorized into two types. The first type is vacuolar cell death that is characterized by the elimination of contents by autophagy events. The second type is necrosis that arises normally due to the rupture of the cell membrane as a consequence of any abiotic stress [81]. *SPL6 (SQUAMOSA PROMOTER BINDING PROEIN LIKE 6)* regulated the panicle development by controlling the expression of an endoplasmic stress signal transducer (IRE1) INSITOL REQUIRING ENZYME [82]. The mutant plants that were deficient for *SPL6* have revealed boosted activation of IRE1 in endoplasmic reticulum and have displayed the degenerated panicle. *OsCIPK31 (CALCINEURIN B-LIKE PROTEIN-INTERACTING PROTEIN KINASES)* is involved in the panicle development by a possible interaction with the MAPK (MITOGEN-ACTIVATED PROTEIN KINASE) pathway. A mutant harboring a mutation in *OsCIPK31* revealed panicle degeneration of the apical spikelet, and trypan blue staining also revealed an excessive level of (ROS) reactive oxygen species [5]. From these discoveries, it can be concluded that the occurrence of the PCD in apical spikelets causes the panicle degeneration. In addition to this, several factors can induce or repress the PCD through a complex mechanism derived by different phytohormones, endogenous signals, and several biotic/abiotic factors [83]. 

## 7. Role of Abiotic Stresses on Panicle Development

Rice is a thermophilic crop and suitable light and temperature conditions are necessary for panicle development [84]. Extreme temperature can cause the loss in grain yield by 10% with every increase of 1°C [85]. High temperature and heat damage can affect the panicle differentiation, ear development, flowering time, pollination, and maturity of grains [86]. Many studies have shown that high temperature during the reproductive stage inhibits the anther dehiscence, pollen dissemination, stigma receptivity, and pollen tube elongation which ultimately leads to the reduction in yield [87,88,89]. Drought during the meiosis can also cause the significant degeneration in the panicles [90]. A study has also revealed that ozone stress is associated with the level of nitrogen which can increase the rate of spikelet degeneration [91]. Application of nitrogen during the spikelet differentiation stage has been found effective to reduce the degeneration [17]. Use of nitrogen can also increase the number of spikelets per panicle, thereby increasing the number of meristems [92]. As the degeneration occurred due to excessive shortage of water during meiosis, satisfactory irrigation during the panicle differentiation stage also reduced the rate of degeneration [91]. Some cultivation practices, for example, drying of the soil and the planting density can also be optimized in order to reduce the panicle degeneration rate [93,94]. Results of previous studies have suggested that environmental stresses can promote the remobilization of reserves and assimilates to the apical spikelet by interaction with ABA, which in turn can be helpful for better grain filling.

## 8. Role of miRNAs

The role of miRNAs in plant development is very significant, but phase transition, meristem identity, and flowering are worth mentioning [95]. An ancient family of miRNAs171, targeted the transcription factors OsHAM1-4 (HAIRY MERISTSTEM) that regulated the transition of shoot apical meristem to FMs [96]. *OsSPL14* (*SQUAMOSA PROMOTER BINDING PROTEIN-LIKE 14)* expression was regulated by an OsmiR156, and it was considered essential for ideal plant architecture, especially for panicle morphology [97]. Over expression of OsmiR397, whose product is OsLAC (LACCASE-like protein), displayed an increase in the number of panicle branches [56]. In addition, siRNAs (small interfering) and tsaiRNAs (trans-acting) have also been reported to mediate the inflorescence development in rice [98]. MiRNA172 has been widely reported in *Arabidopsis* and maize to control the meristems transitions, and its function has been conserved in many plant species including rice [22]. 

## 9. Molecular and Physiological Mechanism of Panicle Development and Degeneration: A Discussion Based on Our Hypothesized Model

**Figure 1:**
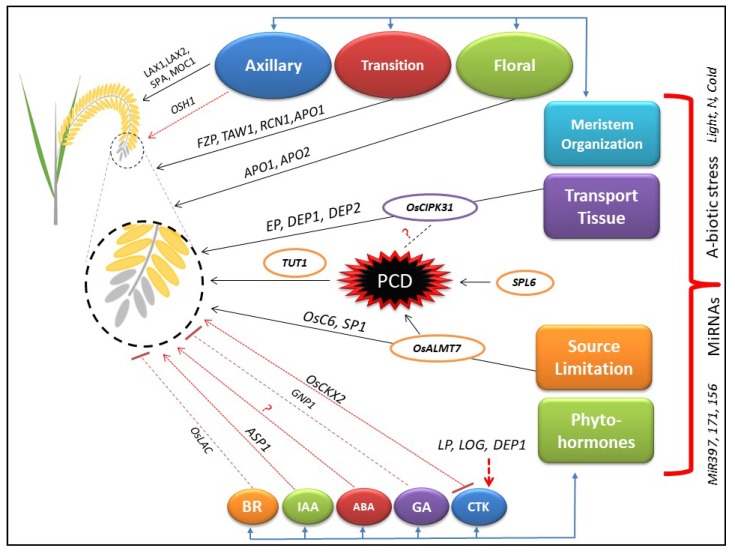
Hypothesized working model for an understanding of the molecular and physiological mechanisms governing panicle development and degeneration. The black arrows show the direct association of specific factors with the panicle degeneration. The dotted red arrows show the regulation of panicle degeneration by controlling the expression of genes that are mentioned above the arrow. The T-head arrows show the negative association of genes with panicle degeneration. The question mark reveals the elucidation mechanism is not reported in studies yet.

Panicle degeneration is affected by a complex network of several genes, biotic, and abiotic factors. We divided the functions of genes based on their physiological mechanisms, for example, meristem organization, transport tissues, source limitation, and phytohormones (Figure 1). The dysfunction and misregulation of panicle development related genes may affect the physiological mechanisms that are ultimately the cause of panicle degeneration. The panicle degeneration is normally induced in the spikelet due to the triggered level of ROS that results into the programmed cell death (PCD). We have enlisted all the reported genes (Table 1) with their known functions to build up a comprehensive understanding about the molecular basis of panicle development and degeneration. The mechanism of meristem organization largely regulates the initiation of inflorescence and specification of lateral branches. The AMs organization in rice panicle is mainly regulated by *LAX1*, *LAX2*, *SPA*, and *MOC1*. The transition meristem controlling genes, for example, *FZP, TAW1*, and *RCN1* regulate the transition of AMs to FMs. Loss of the determinacy of FMs has been reported to affect the panicle architecture by *APO1* and *APO2*. *EP*, *DEP1*, and *DEP2* regulate the panicle architecture, and a mutant showing the loss of function in *OsCIPK31* displays the partial blockage of transport tissues. Most studies of panicle degenerating mutant were principally based on the classical theory of source limitation. *OsALMT7*, *OsC6*, *TUT1*, and *SPL6* cause the panicle degeneration due to constraints in the supply of sugars, fatty acids, and nutrients. The phytohormones are associated with normal panicle development and regulate the physiological mechanisms in a complex way. CTK affects the panicle architecture by negatively regulating the expression of *OsCKX2*. *LP*, *LOG*, and *DEP1* indirectly regulate the expression of CTK that controls the panicle branching in rice. GA plays a positive role in the reproductive meristem development and promotes yield by the expression of *GNP1*. Furthermore, its application decreases the rate of panicle abortion in rice. The role of ABA has not been reported to directly affect the panicle architecture but it seems to have an interaction with ethylene and IAA for regulation of panicle degeneration. IAA plays an important role in the panicle architecture by indirectly regulating the expression of *ASP1* and affects the development of transition meristem. The higher levels of homobrassinolide can be helpful to reduce the abortion of spikelets. The overexpression of miRNAs, for example, OsmiR397, OsmiR171, and OsmiR156 are reported to regulate the ideal morphology of panicle. Abiotic stresses, for example, extreme temperature and low level of nitrogen during the reproductive stage, have also been reported to cause the panicle degeneration in rice species. 

## 10. Conclusions

Panicle degeneration causes a serious loss to the grain yield. Elucidation of the molecular mechanism underlying the panicle degeneration is one of the topical interests of molecular biologists. Source limitation and self-organization processes are the basic physiological mechanism underlying the panicle degeneration. The physiological mechanism underlying panicle degeneration should be considered in order to further validate the concepts of source limitation and self-organization. Explication of molecular mechanisms of *OsCIPK31*, *TUT1*, and *SP6* are recent leading discoveries that made a significant contribution to the knowledge of panicle degeneration. Even though, it was evident from previous studies that ROS are a potential cause of panicle degeneration, most of the mutants were reported without elucidation of the mechanism of ROS production. Future studies should be focused on the crosstalk of phytohormones and their interaction should be counter-checked, as facts about their role in enhancing or suppressing the panicle degeneration are still limited and controversial. Current proposed understanding would be helpful in devising the approaches to cope with the defect of panicle degeneration.

## Figures and Tables

**Table 1:** List of genes, their product, and function required for panicle development.

Gene	Product	Function	References
*APO1/APO2*	F-box protein	floral meristems	[42,45]
*ASP1*	co-repressor	auxin signaling and spikelet development	[35]
*DEP1/DEP2*	PEBP (PHOSPHATIDYLETHALAMINE binding) protein	panicle erectness	[76,77]
*EP2*	Endoplasmic reticulum protein	panicle erectness	[75]
*FZP*	ERF transcription factor	floral meristem establishment	[37]
*GN1*	CYTOKININ OXIDASE	panicle size	[99]
*LAX1*	a bHLH transcription factor	inflorescence architecture	[28]
*LAX2*	a novel nuclear protein	axillary meristems	[29]
*LP*	F-box protein	panicle size	[50]
*MOC1*	GRAS family protein	axillary buds and meristems	[31]
*OSH1*	HOMEOBOX PROTEIN KNOTTED-1-LIKE 6	spikelet development	[33]
*RCN1* and *RCN2*	PEBP (PHOSPHATIDYL-ETHANOLAMINE-BINDING) protein	inflorescence meristem	[41]
*SP1*	PTR transporter	panicle size	[65]
*SPA*	bHLH domain	axillary meristems	[30]
*TAW1*	ALOG (*Arabidopsis* LSH2 and Oryza G1) protein	inflorescence architecture	[39]
*LOG*	CTK- activating enzyme	meristem development	[24]
*SPL6*	IRE1-transducer	control PCD	[82]
*OsCIPK31*	CIPK- protein	panicle development	[5]
*OsALMT7*	ALMT protein	panicle development	[71]
*OsSPL14*	SPL-protein	ideal plant architecture	[97]
*Osc6*	LTP-protein	pollen development	[25]
*TUTOU1*	cAMP/WAVE-like protein	actin organization	[8]
*OsLAC*	LACCASE-like protein	panicle branching	[56]
*GNP1*	GA20ox1	reproductive meristem	[54]
